# Identification of *SOD1* Gene Variants in Mexican Patients with Amyotrophic Lateral Sclerosis

**DOI:** 10.3390/ijms27209157

**Published:** 2026-10-15

**Authors:** Carlos Alberto Gómez-Pérez, Miguel Ángel Ramírez-García, Elizabeth Ruiz-Sánchez, Sandra Romero-Hidalgo, Adriana Ochoa-Morales, Aurelio Jara-Prado, Marisela Méndez-Armenta, Concepción Nava-Ruiz, Petra Yescas-Gómez

**Affiliations:** 1 Departamento de Genética, Instituto Nacional de Neurología y Neurocirugía, Ciudad de México 14269, Mexico; 2 Posgrado en Ciencias Genómicas, Universidad Autónoma de la Ciudad de México, Ciudad de México 03104, Mexico; 3 Laboratorio de Neuroquímica, Instituto Nacional de Neurología y Neurocirugía, Ciudad de México 14269, Mexico; 4 Genómica Computacional, Instituto Nacional de Medicina Genómica, Ciudad de México 14610, Mexico; 5 Laboratorio de Neuropatología Experimental, Instituto Nacional de Neurología y Neurocirugía, Ciudad de México 14269, Mexico

**Keywords:** *SOD1* gene, reactive oxygen species (ROS), Amyotrophic Lateral Sclerosis (ALS), high-resolution melting (HRM), genetic variants, Mexico

## Abstract

Amyotrophic Lateral Sclerosis (ALS) is a rapidly progressing, fatal neurodegenerative disorder that selectively affects motor neurons. Clinical manifestations include progressive muscle weakness, amyotrophy, fasciculations, dysphagia, and fatal respiratory failure. The median survival after symptom onset is typically 2 to 5 years. Despite its severity, the epidemiological profile and genetic landscape of ALS in Latin American populations, particularly in Mexico, remain unclear. In this study, we evaluated the mutational spectrum of the *SOD1* gene in a Mexican cohort of 156 ALS patients. Using high-resolution melting (HRM) analysis and Sanger sequencing, we identified 22 *SOD1* variants in 42 of 156 patients (overall variant carrier frequency, 26.92%). Pathogenic or likely pathogenic (P/LP) variants were found in 21 patients (13.5%). Notably, 5 of the 22 variants were novel, expanding the spectrum of *SOD1* variants. The variant carrier frequency identified in this study is higher than that reported for European populations but similar to frequencies observed in Asian populations. Our results highlight the distinct genetic heterogeneity of ALS in Mexico and demonstrate that HRM is a rapid and effective method for identifying *SOD1* gene variants.

## 1. Introduction

Amyotrophic Lateral Sclerosis (ALS) is a late-onset neurodegenerative disorder with significant clinical, pathological, and genetic heterogeneity [1]. ALS is the most common motor neuron disease (MND) and is characterized by the selective and progressive death of upper motor neurons (UMN) and lower motor neurons (LMN) [2]. Patients with ALS have a median survival of two to five years after symptom onset [3,4]. The annual incidence in Europe is estimated to be between 1.8 and 3.0 per 100,000 people, and the prevalence is estimated to be between 10 and 12 per 100,000 people. However, there is pronounced geographic variation [5,6]. Around 5–10% of cases are familial ALS (fALS), while the remainder are sporadic (sALS) [7,8].

More than fifty genes have been linked to ALS susceptibility and pathogenesis [9]. These genes are often classified by the frequency with which pathogenic variants occur in ALS cohorts, ranging from high-frequency genes, such as *C9orf72*, *SOD1*, *FUS*, *TBK1*, and *TARDBP*, to lower-frequency genes, including *CHMP2B*, *SETX*, *SQSTM1*, and *NEK1*. Many of them are also implicated in frontotemporal dementia (FTD) [10]. Pathogenic variants in these genes can lead to failures in various mechanisms, including vesicular transport, autophagy, cytoskeletal dynamics, mitochondrial function, glutamate-mediated excitotoxicity, alternative mRNA splicing, protein trafficking, and redox dysfunction—ultimately promoting motor neuron degeneration and ALS progression.

Deleterious variants in the Cu/Zn superoxide dismutase gene (*SOD1*, OMIM 147450) constitute the second most common genetic cause of ALS, accounting for ~20% of fALS and ~7% of sALS [11]. This protein is highly conserved throughout evolution [12]. Its primary function is thought to be as a cytosolic and mitochondrial antioxidant enzyme, catalyzing the dismutation of the O_2_^•−^ radical into O_2_ and H_2_O_2_ [13]. *SOD1* gene variants can lead to multiple cellular alterations, including changes in gene expression, abnormal protein interactions, caspase activation, mitochondrial dysfunction, and cytoskeletal alterations [14,15].

Various techniques have been used to study genetic variants in the *SOD1* gene, such as TaqMan assays, PCR, and direct Sanger sequencing. However, only one study has used high-resolution melting (HRM) [11]. This powerful technique distinguishes between different DNA sequences by identifying variations in the melting curves of variant and wild-type alleles [16]. Despite growing worldwide interest, ALS research in Latin America remains limited. In Mexico, data on the genetic contributions to ALS remain limited. This study aims to identify *SOD1* gene variants in sALS and fALS cases through screening with HRM analysis and confirmation with Sanger sequencing.

## 2. Results

**Figure 1:**
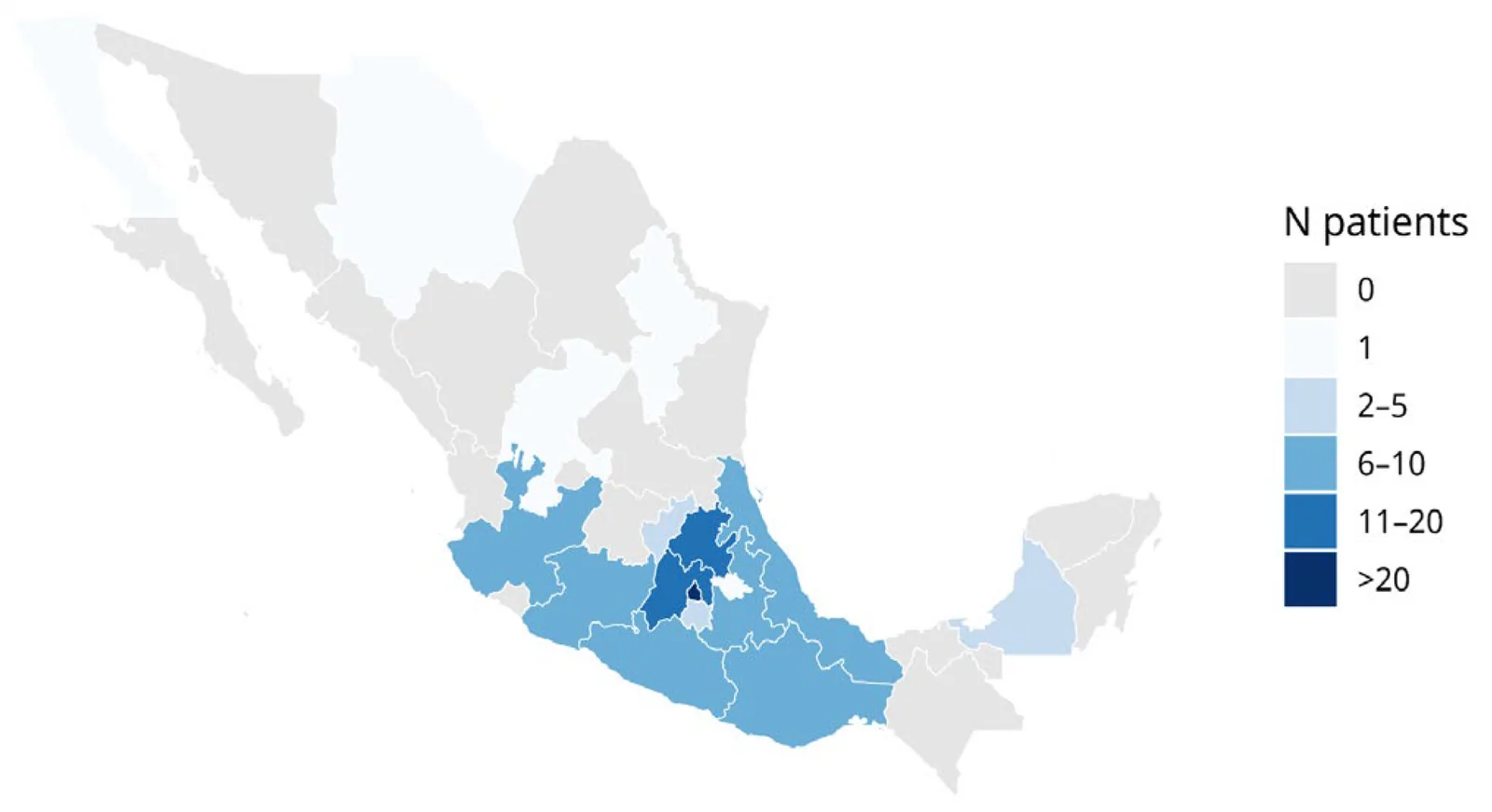
**Geographical distribution of ALS patients in Mexico.** The states of the Mexican Republic with reported cases of ALS included in this study are shown in blue. A higher concentration of cases is observed in the central states of the country, which is attributed to the geographical proximity to the institute where the evaluation was performed (INNNMVS). This distribution does not imply the absence of cases in other regions of the country.

A total of 156 individuals from various regions of the Mexican Republic were analyzed (Figure 1). The largest number of cases came from Mexico City (n = 60). Females accounted for 46.2% (n = 72) of the cases. Regarding diagnostic classification, it was found that 20.5% (n = 32) had a definitive diagnosis of ALS, 7% (n = 11) were classified as probable ALS, 17.30% (n = 27) as probable ALS with laboratory support, and the remaining 55.1% (n = 86) were cases of possible ALS.

Sporadic cases accounted for 90.38% (n = 141), while the remaining 9.61% (n = 15) were familial cases across 14 families. The mean age at diagnosis was 51.56 years (±11.26; range 18–80), and the mean age at symptom onset was 48.51 years (±11.55; range 17–79). The mean time of evolution at the last examination was 47.6 months (±48.2; median 32.5, IQR 21.3–48.0). Concerning clinical presentation, 31.4% (n = 49) exhibited bulbar onset and 60.2% (n = 94) had spinal onset. Among the spinal cases, 59 (62.76%) were brachial, and 35 (37.23%) were crural. Additionally, 13 individuals (8.33%) were identified as having Vulpian-Bernhardt syndrome, which is characterized by exclusive involvement of the cervical segment for at least 12 months and a predominance of lower motor neuron involvement. This condition typically has an asymmetric onset, affecting the proximal muscles more than the distal muscles in 70% of cases in the early stages. Vulpian-Bernhardt syndrome accounts for 2–11.4% of ELA motor phenotypes. Due to its longer survival time of 5–10 years, it is clinically significant [17]. The estimated minimum frequency of ALS in Mexico City was 0.65 cases per 100,000 individuals.

### 2.1. HRM Scanning and Sequencing Confirmation

**Figure 2:**
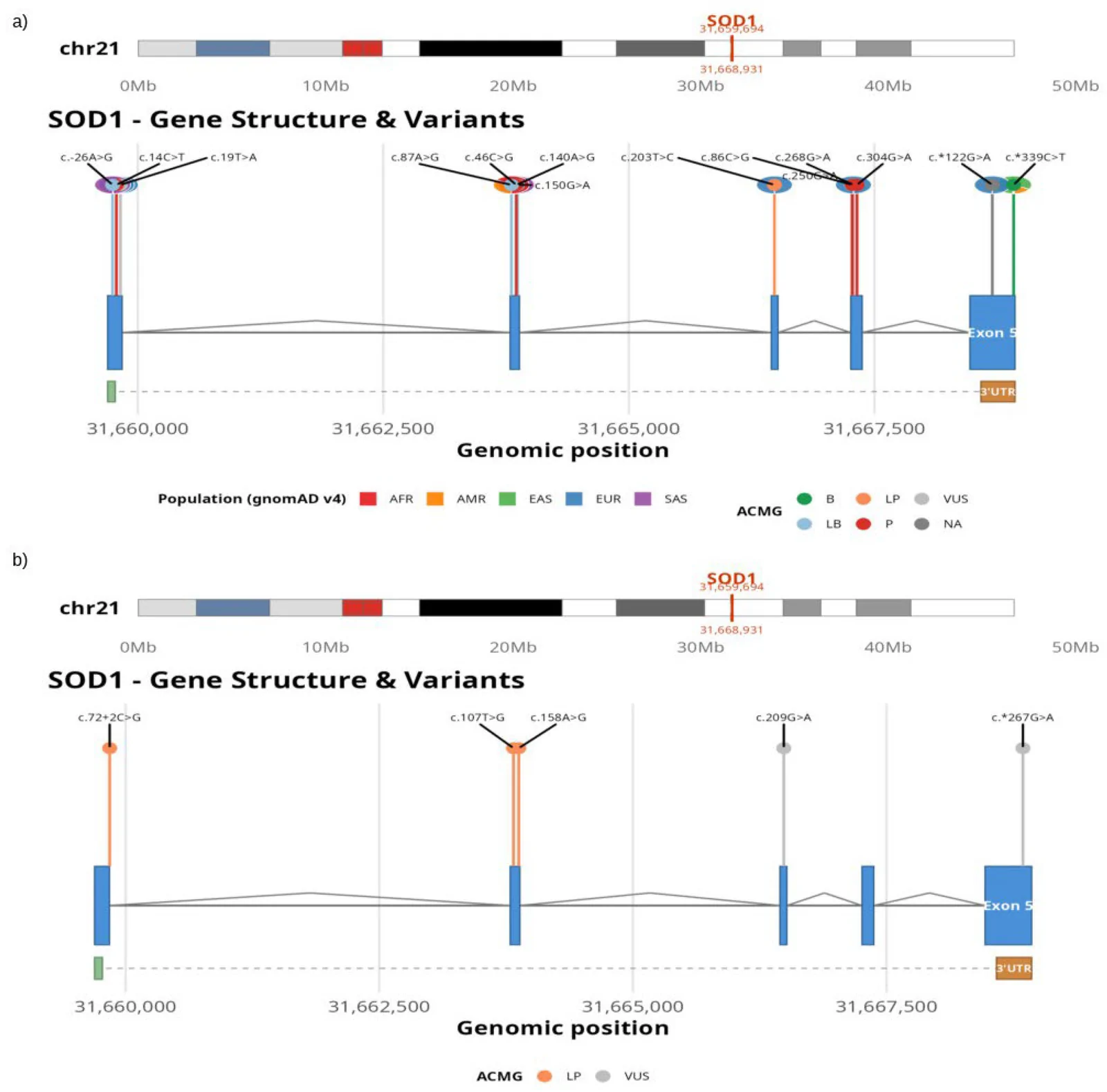
**The *SOD1* genetic variant landscape in a Mexican ALS cohort.** (**a**) Previously reported *SOD1* variants identified in Mexican ALS patients mapped to the genomic structure of transcript NM_000454.5 (GRCh38). Lollipop colors indicate ACMG classification (P: red, LP: orange, VUS: gray, LB: light blue, B: green). Population allele frequencies from gnomAD v4 are shown as proportional pie charts (AFR: red, AMR: orange, EAS: green, EUR: blue, SAS: purple). Variants absent from gnomAD display only the ACMG classification point. (**b**) Five novel *SOD1* variants identified for the first time in a Mexican ALS cohort. None of these variants are reported in gnomAD v4, ClinVar, or previous ALS literature, suggesting they may represent population-specific variants in admixed Latin American individuals. Chromosomal ideogram (top) shows the location of *SOD1* on chromosome 21.

A total of 22 changes (Figure 2) to the melting curves were identified in the different exons of the *SOD1* gene in 42 out of 156 individuals analyzed (26.92%). In contrast, the controls showed tight clustering for all fragments. This allowed suggestive samples that might have carried genetic alterations to be clearly distinguished from one another. Corroboration using Sanger sequencing confirmed at least a genetic variant in all samples with abnormal melting profiles (42/42).

### 2.2. Variants Identified in the SOD1 Gene

Of the 42 individuals with ALS who carry genetic variants, 36 were sporadic cases, and six were familial cases (5 families). Among these individuals, 41 carried at least one of the 17 previously reported variants: five pathogenic (P), three likely pathogenic (LP), four of uncertain significance (VUS), three likely benign (LB), and two benign (B) (Table 1). Twenty-one individuals (20 sporadic and one familial) carried at least one of five novel variants, including three missense variants (c.107T>G, c.158A>G and c.209G>A) and two splicing variants (c.72+2C>G, c.*267G>A) (Table 1).

### 2.3. Validation of SOD1 Gene Novel Variants and Clinical Presentation

The bioinformatics predictors HOPE, DUET, MutPred2, CADD, and MutationTaster were used to analyze the potential functional and structural impacts of the identified variants. In contrast, the assessment of the possible functional impact of the splicing variants was performed with Human Splicing Finder, SpliceAI, Ex-SKIP, CADD, and MutationTaster. Table 2 summarizes these results.

### 2.4. Identification of VUS in the SOD1 Gene

The c.86C>G (p.Pro29Arg) variant was identified in six unrelated patients (cases 19, 20, 21, 22, 23, and 25). These patients predominantly presented with spinal forms and a crural subphenotype. Two of these patients exhibited FTD features (cases 19 and 21).

**Figure 3:**
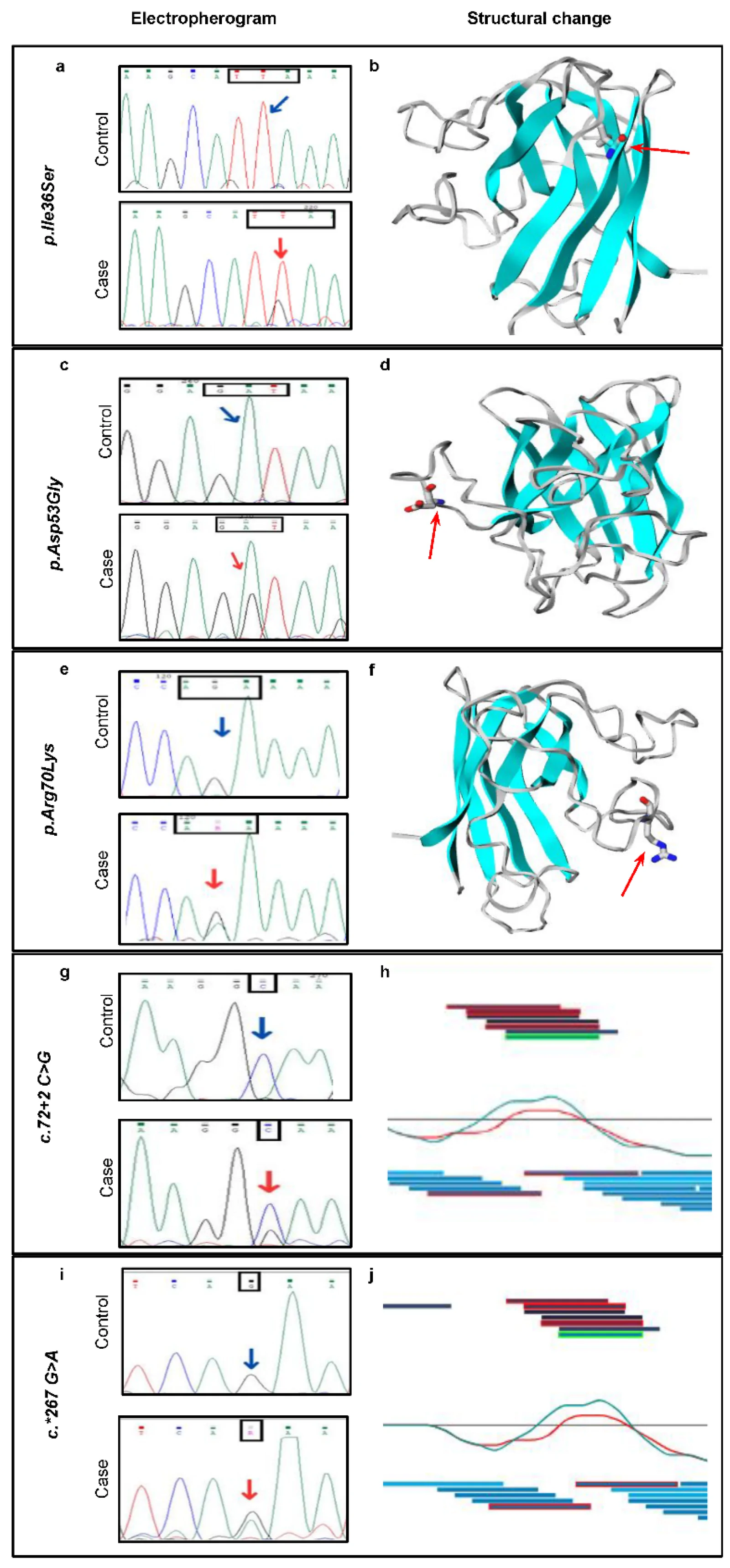
**Electropherograms and inferred structural/splicing impacts on the novel *SOD1* variants.** The left panels (**a**,**c**,**e**,**g**,**i**) show a comparison of Sanger sequencing electropherograms between controls and cases. The blue arrow indicates the baseline, while the red arrow indicates the observed change. The right panels (**b**,**d**,**f**) show the predicted structural changes in the SOD1 protein (c.107T>G, c.158A>G, c.209G>A). The red arrows show the position of the amino acid change. Panels (**h**,**j**) correspond to the possible functional effects of the splicing variants (c.72+2C>G and c.*267G>A). Blue bars represent exonic splicing enhancer (ESE) and exonic splicing silencer (ESS) elements. Green highlighting indicates the creation of new elements, while red highlighting indicates the disruption or cleavage of existing elements, suggesting a potential effect on *SOD1* gene expression.

The c.209G>A (p.Arg70Lys) variant was detected in three patients (cases 31, 32, and 33; see Figure 3e,f, as well as Table 2). Two of these patients presented with Bulbar onset (cases 32 and 33), while the others presented with spinal onset and a brachial subphenotype.

### 2.5. Identification of LP in the SOD1 Gene

The c.107T>G (p.Ile36Ser) variant was detected in two unrelated patients (cases 23 and 25), as shown in Figure 3a,b and Table 2. Both patients presented with spinal onset and the crural subphenotype. Similarly, both patients carried the p.Pro29Arg variant. The c.158A>G (p.Asp53Gly) variant was detected in four patients (cases 19, 21, 22, and 25) (see Figure 3c,d, as well as Table 2). Three of these patients had a spinal onset with a crural subphenotype (cases 19, 22, and 25), and one had a Bulbar onset (case 21).

### 2.6. Identification of Splice Variants in the SOD1 Gene

The c.72+2C>G splice variant (see Figure 3g,h, as well as Table 2) was identified in intron 1–2 in three females with bulbar onset disease (cases 2, 5, and 6). These individuals experienced early disease onset (ages 37–47) and slow disease progression. Lastly, the c.*267G>A variant (see Figure 3i,j, as well as Table 2) was identified in 11 individuals (six females and five males) who exhibited heterogeneous forms and ages of disease onset.

## 3. Discussion

ALS is a rare neurodegenerative disorder with rapid progression and a fatal outcome. Deleterious variants of the *SOD1* gene are a common genetic cause of both familial and sporadic ALS.

In our ALS cohort, the age of onset and the proportion of bulbar and spinal forms were similar to those of a previous Mexican study [18]. However, the latter study estimated a mean diagnostic delay of 12 months. In our cohort, the mean delay was 36.2 ± 36.0 months (median 24, IQR 12–48; 95% CI of the mean 30.5–42.0; n = 153). As individual-level data were unavailable for the comparator study, a formal statistical comparison could not be conducted, and this apparent difference should be interpreted with caution. Comparing our clinical findings with those of other international cohorts, notable differences are observed. In the studies by Raymond et al. [19] and, Tiawijit et al. [20], the predominant clinical phenotype is reported to be spinal (72.0% and 80%, respectively), followed by bulbar onset (22.1% and 20%, respectively), a distribution that matches that observed in our cohort. Interestingly, Tiawijit et al. report a mean age of onset of 53.2 years in their Thai cohort, which is similar to the figure observed in our study. Other cohorts reported shorter diagnostic delays than the mean of 36 months observed in our study [19,20,21], although these comparisons are descriptive. Another notable difference was the proportion of fALS: 9.61% in our cohort versus the 13–20% in other studies [21].

To our knowledge, this is the second study worldwide to use HRM analysis to screen ALS patients for *SOD1* gene variants. Akimoto et al. [11] reported a sensitivity of 94.7%. In our study, Sanger sequencing revealed that all samples with abnormal melting profiles carried a genetic variant (42/42). However, the sensitivity of HRM could not be determined, since samples with normal profiles were not sequenced. Our results also support the use of HRM screening followed by Sanger sequencing as a cost-effective, rapid strategy. Our findings are consistent with those of other studies that have used HRM to identify variants in clinically relevant genes [22,23,24,25,26,27,28,29].

The frequency of *SOD1* gene variants associated with ALS differs between populations. For instance, a European cohort found 11% of gene variants [30], whereas Asian populations (25.1–35.1%) [31], which is similar to our overall variant carrier frequency (26.92%, 42/156). This similarity may reflect a shared ancestral origin, with roots in Native American and East Asian populations [32,33]. This origin has been shaped by migratory and historical processes over millennia. For example, genetic variants associated with metabolic risk are highly prevalent in Mexico and show similar frequencies in the Asian population [34]. However, the frequency of P/LP variants in our cohort was 13.5% (21/156), and half of the carriers (21/42) had only VUS, LB, or B variants. This distinction is important in order to avoid overestimating the genetic burden of *SOD1* in the Mexican population. Furthermore, more than half of our cohort (55.1%, n = 86) was classified as possible ALS according to the revised El Escorial criteria [35], which may have influenced the reported frequencies. Lower diagnostic certainty increases the likelihood of phenotypic heterogeneity and of including ALS mimics [36,37]. Since ALS mimics are not expected to have an increased frequency of SOD1 pathogenic variants, including them would likely lower the P/LP frequency rather than increase it. In contrast, the overall carrier frequency is largely unrelated to diagnostic certainty. Therefore, our frequencies should be interpreted as representative of the entire clinically selected cohort attending a tertiary referral center, rather than of genetically confirmed ALS cases. Furthermore, the comparator cohorts may include a higher proportion of definite or probable cases. It is also unclear whether their frequencies refer to pathogenic variants only or to all detected variants. Therefore, cross-population comparisons should be interpreted with caution.

Of the 36 sALS cases, 35 carried at least one of 15 previously reported variants: three P (c.140A>G, c.203T>C, and c.268G>A), three LP (c.250G>A, c.304G>A, and c.425G>A), five VUS (c.201T>C, c.46C>G, c.58A>C, c.86C>G, and c.*122G>A), three LB (c.-26A>G, c.87A>G, and c.150G>A), and one B (c.*339C>T). Twenty individuals carried at least one of the five novel variants described below (Table 1). Of these, 19 also carried a previously reported variant, and only one (case 32) carried a novel variant alone (c.209G>A). All six fALS cases carried at least one of six previously reported variants: four P (c.140A>G, c.14C>T, c.19T>A, and c.268G>A), one VUS (c.*122G>A), and one B (c.*339C>T). Only one individual (case 1) also carried the novel variant c.*267G>A.

A total of 21 out of 42 individuals with *SOD1* variants (50%) were found to carry at least one P/LP variant. Thirteen of these individuals carried a variant that had previously been reported, while eight carried only novel LP variants (c.72+2C>G, c.107T>G or c.158A>G). P/LP variants were more prevalent in fALS cases than in sALS cases (6/15, 40%, 95% CI 16.3–67.7 vs. 15/141, 10.6%, 95% CI 6.1–16.9; Fisher’s exact test, *p* = 0.0066). However, this result should be interpreted with caution due to the small number of familial cases and wide confidence interval. Previously reported VUS were more frequent in sALS (21/36, 58%) than in fALS (1/6, 17%), whereas previously reported LB/B variants were found in 14/36 sALS cases (39%) and one fALS case (1/6; 17%). Novel variants were present in 20 sALS cases (56%) and one fALS case (17%). With the exception of case 32, novel variants co-occurred with a previously reported variant.

### 3.1. P and LP Variants

The c.14C>T (p.Ala5Val) P variant was identified in an individual presenting with Bulbar onset ALS. A previous report describes a family carrying this variant. In that report, the affected carriers presented with spinal onset with signs of LMN involvement and a survival time of 20 months [38]. Furthermore, this variant has been observed to affect Cu^2+^ ion binding at a functional level [39]. Meanwhile, the c.304G>A (p.Asp102Asn) LP variant was identified in a 55-year-old female patient with a bulbar phenotype. This variant has been identified in sALS and fALS cases in the European population [40,41]. It has also been associated with an early age of onset.

Through in silico prediction, we propose that the novel c.158A>G (p.Asp53Gly) variant (identified in 4 patients) induces structural destabilization, impairing the critical process of protein oligomerization. This alteration may promote the formation of non-native oligomeric species, particularly trimers. Trimers have recently been associated with increased cytotoxicity and neuronal cell death in ALS models [42,43]. On the other hand, the p.Ile36Ser variant is inferred to cause significant destabilization through a reduction in local hydrophobicity, which could lead to aberrant polar contacts and destabilization of the native SOD1 structure; however, this variant continues to be classified as LP. Although the mechanisms differ, the p.Asp90Val variant has been described to alter the hydrophobic balance of the SOD1 protein [44,45], highlighting the crucial role of physicochemical properties in maintaining protein stability and functionality.

### 3.2. Splice Variants

The c.72+2C>G variant affects the canonical +2 position of the donor splice site. Such variants can cause exon skipping or activate cryptic splice sites, resulting in the inclusion or deletion of exonic or intronic segments [46,47]. This has been observed in other ALS-related genes, such as *SETX*, *TARDBP* and *OPTN* [48,49,50]. For the c.72+2C>G variant, the predictors were concordant. SpliceAI predicted both donor loss (Δ = 0.92) and a 57 bp donor gain upstream, whereas Pangolin predicted a loss of splicing (Δ = 0.72). The SpliceAI-10k calculator predicted the use of a cryptic donor site in exon 1, resulting in the deletion of 55 bp in the coding sequence and a frameshift mutation. Although toxic gain of function is the main pathogenic mechanism of SOD1-related ALS and loss of function is not an established disease mechanism for this gene, loss of function cannot be ruled out as a contributing factor. This is because the removal of mutant SOD1 from the nucleus has been suggested to impair its protective role in SOD1^G93A^ models [51]. A combination of PVS1 (PVS1_Strong) and PM2 (PM2_Moderate) was sufficient for an LP classification of c.72+2C>G, whereas HSF predicted ESE/ESS alterations for c.-26A>G, c.*122G>A, c.*267G>A and c.*339C>T. However, SpliceAI and Pangolin predicted no impact for these variants (Δ ≤ 0.01 and Δ ≤ 0.08, respectively). Due to the limited specificity of motif-based predictions, the splicing effect was deemed unsupported for these variants and their classification was not modified.

### 3.3. Variants of Uncertain Significance

The c.46C>G (p.Gln16Glu) variant was identified in three female individuals with Bulbar onset forms. Upon analyzing the potential impact of this mutation, we found that the difference in charge between the wild-type and mutant residues could affect the interaction between the protein and other molecules, as well as induce repulsion between neighboring residues. This variant was previously reported by Ruffo et al. [41]. Additionally, the novel p.Arg70Lys variant is a substitution of two basic amino acids that preserves the positive charge (Figure 3e,f). Our analysis yielded contrasting results: some predictors suggested a deleterious effect, while others indicated a minor impact of this substitution. Due to the absence of additional ACMG criteria supporting a clear pathogenic role (such as PP2 or PP3) and the results of the used predictors, we conclude that the p.Arg70Lys mutation should remain classified as a VUS. This emphasizes the need for further functional or segregation studies to clarify its clinical relevance.

### 3.4. Co-Occurrence of SOD1 Gene Variants

Interestingly, we identified patients with co-occurring *SOD1* gene variants. One patient had six variants (case 23, Table 1); two patients had four variants each (cases 1 and 25, Table 1); and five patients had three variants each. Additionally, 11 cases exhibited co-occurrence of the c.*122G>A and c.*267G>A variants. The presence of multiple genetic variants in the same individual suggests that these variants may have combined effects on *SOD1* function. These effects may include altered protein stability, dimerization, and splicing regulation as has been proposed by Zhang et al. [52] regarding the co-occurrence of variants in the same gene or in other genes associated with early-onset Alzheimer’s disease. These possible additive effects underscore the necessity of further functional studies to investigate the pathogenic relevance of these variant combinations.

Recently, cases of co-occurrence in different ALS-related genes have been reported. For instance, Yilmaz et al. [53] described an individual with sporadic ALS who carried four genetic variants: p.Gly287Ser in *TARDBP*, p.Glu102* in *NEK1*, p.Gly1149Val in *ERBB4*, and p.Arg339Trp in *VEGFA*. Similarly, Dong et al. [54] described fALS cases with three genetic variants: *SOD1* (p.V48A), *OPTN* (p.A433V), and *TBK1* (p.R573H). These results support the hypothesis proposed by Lattante et al. [55] that the genetic architecture of ALS cases is more complex than the Mendelian inheritance patterns suggest.

### 3.5. Strengths, Limitations and Perspectives

Based on the results obtained, we consider that our study had a robust sample of ALS cases; however, we recognize that there are limitations related to their diagnostic classification, since most cases were classified as possible ALS. Although the HRM technique is low-cost, it requires more time and processing than massive sequencing technologies. However, these technologies are still too expensive and inaccessible for most research laboratories. In the future, HRM could be used to evaluate *SOD1* gene methylation as a potential ALS mechanism. Similarly, identifying novel genetic variants in other populations is interesting and suggests that this disease may be related to ancestry. Therefore, studying the effect of co-occurrence with other *SOD1* gene variants would be informative.

## 4. Materials and Methods

### 4.1. Patients and Samples

This study enrolled Mexican patients diagnosed with ALS between 2016 and 2020 in the Instituto Nacional de Neurología y Neurocirugía Manuel Velasco Suárez (INNNMVS) in Mexico City. Experienced neurologists and neurogeneticists at our institution examined patients and made diagnoses using the revised El Escorial criteria [35]. The study protocol (protocol code 262111, approved in 5 March 2015) adhered to the Declaration of Helsinki and was approved by the institutional research and ethics committees. All participants provided written informed consent before peripheral blood collection.

### 4.2. DNA Extraction

Genomic DNA was extracted from peripheral blood samples taken from patients with clinical ALS, using a QIAamp DNA Blood Midi Kit (QIAGEN, Hilden, Germany) according to the manufacturer’s instructions. We analyzed the concentration and purity of the DNA by spectrophotometry using a NanoDrop ND-2000 (Thermo Fisher Scientific, Waltham, MA, USA). Next, we prepared 50 ng/µL DNA aliquots and ran them on 1% agarose gels to assess the integrity of the DNA. Samples that did not meet the required concentration or integrity criteria were excluded from the analysis.

### 4.3. Screening of SOD1 Gene Variants by PCR-HRM

PCR primers covering the five exons of *SOD1* were designed with Primer3Plus (https://www.primer3plus.com). PCR-HRM was carried out on a Rotor-Gene RG-6000 (Corbett Research, Sydney, Australia) in 16 µL reactions containing 1.6 µL 10× PCR buffer, 0.8 µL KB Extender, 1 µL each of forward and reverse primers (10 ng/µL), 0.8 µL SYTO-9 (1:100; Thermo Fisher Scientific, Waltham, MA, USA), 0.48 µL MgCl_2_ (50 mM), 0.32 µL dNTP mix (10 mM), 0.064 µL Platinum™ Taq DNA Polymerase (Invitrogen, Waltham, MA, USA), 2 µL genomic DNA (50 ng/µL), and nuclease-free water to volume. Thermal cycling consisted of an initial denaturation at 95 °C for 3 min, followed by 35 cycles of 95 °C for 10 s, annealing at 56–66 °C (primer-dependent) for 40 s, and extension at 72 °C for 10 s. Melting curves were recorded from 75 to 95 °C with Rotor-Gene Q-Series software v.1.7 (QIAGEN, Hilden, Germany). Each sample was analyzed in duplicate, and those samples showing abnormal melting patterns were retested in an independent duplicate assay. Samples that replicated these abnormal patterns were considered positive and verified by Sanger sequencing.

### 4.4. Sanger Sequencing and Bioinformatics Analysis

Samples were sequenced from individuals suspected of carrying the genetic variant identified in the HRM analysis. Sanger sequencing was performed on an ABI 3130 Genetic Analyzer (Applied Biosystems, Foster City, CA, USA). Electropherograms were analyzed using Chromas software v.2.6.6.

After variant identification, they were classified according to ACMG criteria. All identified variants were organized according to whether they were previously known or novel. We analyzed the possible functional effects of the splicing variants using the Human Splicing Finder (https://genomnis.com/hsf, accessed on 12 June 2026), SpliceAI (https://spliceailookup.broadinstitute.org/), which provides SpliceAI, Pangolin and SpliceAI-10k calculator predictions, and Ex-SKIP tools. Similarly, a predictive analysis of the functional effect at the structural level was performed for the missense variants using HOPE v.1.1.1, DUET (https://biosig.lab.uq.edu.au/duet/stability, accessed on 7 July 2026), MutPred2 (https://mutpred2.mutdb.org/index.html, accessed on 7 July 2026), CADD v.1.7, and MutationTaster (https://www.genecascade.org/index.html, accessed on 7 July 2026) predictors, using the MANE Select transcript NM_000454.5 (GRCh38). It should be noted that the analysis using the latter platform employed a monomer of protein (PDB: 1BA9).

### 4.5. Statistical Analysis

The diagnostic delay was defined as the interval between the onset of symptoms and diagnosis. It was calculated using the age at symptom onset and diagnosis, and expressed in months. Continuous variables are reported as the mean ± standard deviation or the median (interquartile range). The 95% confidence intervals for the mean were calculated using the t-distribution. Categorical variables were compared using Fisher’s exact test and 95% confidence intervals for proportions were calculated using the Clopper–Pearson method. The only formal hypothesis test performed was a comparison of the frequency of P/LP variants between familial and sporadic cases. All patients in each group were used as the denominator, so no correction for multiple testing was applied. A two-sided *p*-value < 0.05 was considered significant. Patients with missing data were excluded from the corresponding analyses. Comparisons with published cohorts were descriptive. Analyses were performed using R v.4.5.2.

## 5. Conclusions

This study provides the first characterisation of the *SOD1* mutation profile in Mexican patients with ALS, achieved through the use of HRM and Sanger sequencing. The overall frequency of variant carriers was found to be 26.92% (42/56), while the frequency of P/LP variants was found to be 13.5% (21/156). As comparator cohorts do not always specify whether their frequencies refer to P variants only or to all detected variants, direct comparisons should be interpreted with caution. Twenty-two variants were identified, five of which were novel, expanding the known genetic landscape of ALS in an underrepresented population. These results support the use of HRM followed by Sanger sequencing as a rapid and cost-efficient screening strategy, although the sensitivity of this approach could not be determined. These findings may inform genetic counselling and the incorporation of molecular tools into ALS diagnostic frameworks.

## Figures and Tables

**Table 1:** Genetic variants identified in ALS Mexican patients.

Patient	Sex	Age (Years)	AOO (Years)	ALS Type	*SOD1* Variant (c.DNA)	SOD1 Variant (Protein)	Variant Type	Significance	EDC	Clinical Features	Time of Evolution at Last Examination (Months)
1	m	54	52	F	c.268G>A c.*339C>T c.*122G>A c.*267G>A	p.Ala90Thr NA NA NA	Missense Splice Variant Splice Variant Splice Variant	P B VUS VUS	1	Spinal onset with crural subphenotype	48
2	f	58	46	S	c.-26A>G c.72+2C>G c.*339C>T	NA NA NA	Splice Variant Splice Variant Splice Variant	LB LP B	4	Bulbar onset	108
3	m	20	17	S	c.*339C>T	NA	Splice Variant	B	4	Spinal onset with crural subphenotype	132
4	f	58	57	S	c.250G>A	p.Asp84Asn	Missense	LP	4	Bulbar onset	18
5	f	49	47	S	c.46C>G c.72+2C>G	p.Gln16Glu NA	Missense Splice Variant	VUS LP	4	Bulbar onset with crural subphenotype	24
6	f	40	37	S	c.46C>G c.72+2C>G	p.Gln16Glu NA	Missense Splice Variant	VUS LP	4	Bulbar onset with crural subphenotype	60
7	m	-	49	F	c.268G>A	p.Ala90Thr	Missense	P	3	Spinal onset with crural subphenotype	108
8	f	44	42	S	c.*122G>A	NA	Splice Variant	VUS	4	Spinal onset with crural subphenotype	24
9	f	41	36	S	c.-26A>G	NA	Splice Variant	LB	4	Spinal onset with crural subphenotype	60
10	f	60	45	F	c.19T>A	p.Cys7Ser	Missense	P	4	Spinal onset with crural subphenotype	180
11	m	34	32	S	c.140A>G	p.His47Arg	Missense	P	1	Bulbar onset with Vulpian-Bernhardt syndrome	22
12	m	32	27	F	c.140A>G	p.His47Arg	Missense	P	4	Spinal onset with crural subphenotype	67
13	m	61	55	S	c.*122G>A c.*267G>A	NA NA	Splice Variant Splice Variant	VUS VUS	1	Spinal onset with crural subphenotype and parkinsonism	74
14	f	40	39	S	c.-26A>G	NA	Splice Variant	LB	4	Bulbar onset	22
15	f	59	56	S	c.*339C>T	NA	Splice Variant	B	4	Spinal onset with brachial subphenotype	36
16	m	38	36	S	c.*122G>A c.*267G>A	NA NA	Splice Variant Splice Variant	VUS VUS	4	Spinal onset with brachial subphenotype	24
17	f	36	31	S	c.268G>A c.*122G>A c.*267G>A	p.Ala90Thr NA NA	Missense Splice Variant Splice Variant	P VUS VUS	3	Spinal onset with crural subphenotype	68
18	m	60	50	S	c.150G>A	p.Glu50=	Synonymous	LB	1	Bulbar onset	163
19	f	72	71	S	c.86C>G c.158A>G	p.Pro29Arg p.Asp53Gly	Missense Missense	VUS LP	4	Spinal onset with crural subphenotype and FTD	12
20	m	64	64	S	c.86C>G c.87A>G	p.Pro29Arg p.Pro29=	Missense Synonymous	VUS LB	1	Spinal onset with crural subphenotype	16
21	f	64	64	S	c.86C>G c.158A>G	p.Pro29Arg p.Asp53Gly	Missense Missense	VUS LP	3	Bulbar onset and FTD	24
22	f	44	42	S	c.86C>G c.87A>G c.158A>G	p.Pro29Arg p.Pro29= p.Asp53Gly	Missense Synonymous Missense	VUS LB LP	2	Spinal onset with crural subphenotype	24
23	f	80	79	S	c.86C>G c.87A>G c.107T>G c.*122G>A c.*267G>A c.*339C>T	p.Pro29Arg p.Pro29= p.Ile36Ser NA NA NA	Missense Synonymous Missense Splice Variant Splice Variant Splice Variant	VUS LB LP VUS VUS B	4	Spinal onset with crural subphenotype	24
24	f	50	48	S	c.58A>C	p.Asn20His	Missense	VUS	3	Spinal onset with crural subphenotype	60
25	m	50	49	S	c.86C>G c.87A>G c.107T>G c.158A>G	p.Pro29Arg p.Pro29= p.Ile36Ser p.Asp53Gly	Missense Synonymous Missense Missense	VUS LB LP LP	4	Spinal onset with crural subphenotype	46
26	f	18	17	S	c.*122G>A c.*267G>A	NA NA	Splice Variant Splice Variant	VUS VUS	1	Bulbar onset with brachial subphenotype	12
27	f	24	23	S	c.268G>A	p.Ala90Thr	Missense	P	2	Juvenile case of spinal onset with crural subphenotype	9
28	m	61	60	S	c.425G>A	p.Gly142Glu	Missense	LP	2	-	12
29	m	40	37	S	c.*122G>A c.*267G>A	NA NA	Splice Variant Splice Variant	VUS VUS	2	Spinal onset with brachial subphenotype	46
30	m	43	41	S	c.-26A>G	NA	Splice Variant	LB	1	Spinal onset with brachial subphenotype	36
31	m	53	51	S	c.201T>C c.203T>C c.209G>A	p.Pro67= p.Leu68Pro p.Arg70Lys	Synonymous Missense Missense	VUS P VUS	2	Spinal onset with brachial subphenotype	23
32	m	62	61	S	c.209G>A	p.Arg70Lys	Missense	VUS	3	Bulbar onset with crural subphenotype	17
33	f	71	69	S	c.46C>G c.209G>A	p.Gln16Glu p.Arg70Lys	Missense Missense	VUS VUS	2	Bulbar onset	24
34	f	-	52	S	c.304G>A	p.Asp102Asn	Missense	LP	1	Bulbar onset	33
35	f	49	48	S	c.*339C>T	NA	Splice Variant	B	4	Spinal onset with brachial subphenotype	7
36	f	70	69	S	c.*122G>A c.*267G>A c.*339C>T	NA NA NA	Splice Variant Splice Variant Splice Variant	VUS VUS B	4	Bulbar onset	4
37	m	-	55	F	c.14C>T	p.Ala5Val	Missense	P	4	Bulbar onset	17
38	m	68	62	S	c.*339C>T	NA	Splice Variant	B	4	Spinal onset	144
39	m	62	60	S	c.*122G>A c.*267G>A	NA NA	Splice Variant Splice Variant	VUS VUS	3	Spinal onset with crural subphenotype	29
40	f	27	24	S	c.*122G>A c.*267G>A	NA NA	Splice Variant Splice Variant	VUS VUS	1	Spinal onset with brachial subphenotype	52
41	m	47	41	F	c.140A>G	p.His47Arg	Missense	P	4	-	87
42	f	46	42	S	c.*122G>A c.*267G>A	NA NA	Splice Variant Splice Variant	VUS VUS	4	-	48

Female (f); Male (m), Age of onset (AOO); Familial (F); Sporadic (S); Pathogenic (P); Likely Pathogenic (LP); Variant of uncertain significance (VUS); Likely Benign (LB); Benign (B); El Escorial diagnostic classification (EDC): 1, definite; 2, probable; 3, probable with laboratory support; 4, possible; missing data (-).

**Table 2:** In silico predictions and ACMG classification of the novel *SOD1* variants.

	Variant	ACMG Classification	ACMG Criteria	DUET	HOPE	Mutation Taster	CADD PHRED Score	MutPred2 Score
**Missense variant**	p.Pro29Arg *	VUS	PM1, PM2	ΔΔG = −0.4 kcal/mol	Disturbs domains involved in interactions with other molecules.	Benign	2.508	0.233
	p.Ile36Ser	LP	PM2, PM1, PP2, PP3	ΔΔG = –3.44 kcal/mol	Reduces local hydrophobicity and may create novel polar contacts.	Deleterious	14.7	0.841
	p.Asp53Gly	LP	PM2, PM1, PP2, PP3	ΔΔG = +0.75 kcal/mol	May compromise oligomerization.	Deleterious	21.2	0.907
	p.Arg70Lys	VUS	PM2, PM1	ΔΔG = –0.40 kcal/mol	Preserves the positive charge of the wild-type residue; possible loss of intermolecular interactions.	Deleterious	14.3	0.046
	**Variant**	**ACMG Classification**	**ACMG Criteria**	**HSF**	**SpliceAI**	**Mutation Taster**	**CADD PHRED**	**EX-SKIP**
**Splice variant**	c.72+2C>G	LP	PM2, PVS1	Donor site alterations	Donor loss 0.92 Donor gain 0.44	Deleterious (Splice site lost)	23.8	EIE 6.3279 NI-ESE 0.9725
	c.*267G>A	VUS	BP7, PM2	ESE and ESS alterations	Δ ≤ 0.01	Benign	3.446	NI-ESE 0.4621

Variant of uncertain significance (VUS); Likely Pathogenic (LP); Exonic Splicing Enhancer (ESE); Exonic Splicing Silencer (ESS); Exon-Identity Element (EIE); Neighborhood Inference Exonic Splicing Enhancer (NI-ESE). * Although p.Pro29Arg (c.86C>G) has been reported previously, we found no published description of the clinical phenotype of carriers. Its only available classification derives from an in silico reclassification of existing *SOD1* variants. We therefore included it in the in silico analysis to provide a phenotype-linked assessment.

## Data Availability

The original contributions presented in this study are included in the article. Further inquiries can be directed to the corresponding author.
